# On Meat and Milk

**Published:** 1875-11

**Authors:** 


On Meat and Milk.—The *Doctor* says: M. Leven, who has investigated, with much patience, various problems involved in the digestion of food, has drawn from his experiments some practical conclusions which deserve the attention of all practitioners. He says that, in order to ascertain the digestibility of food, we must find out not only what is its aptitude to undergo the action of the digestive juices, but also what effect the food produces on the mucous membrane.

We must know in what part of the digestive tube the food is elaborated, whether in the stomach or in the intestine. Thus, meat stays a long time in the stomach, and milk a very short time. So that, if we have to treat an ulcer in the stomach, we can easily understand how important it is not to give meat, which excites the contraction of the muscular fibres of the stomach, and the circulation of the mucous membrane, in order to avoid returns of hæmatemesis.

Milk, on the contrary, will be preferred; it is an inert substance, which leaves the stomach to repose, and glides softly through the pylorus without any effort of the organ. It has been said, in such cases, that milk is more digestible than meat; but are we authorized to make this statement? No one has measured the time necessary for milk to pass into the circulation. How many hours does it take to emulsionize fat, and to render caseine absorbable? Does milk pass into the blood quicker than meat? That is probable; but we cannot, and ought not, to establish any comparison between the digestibility of these two substances. It does not take place on the same spot, and we have to do with perfectly different substances, between which we ought to make no comparison. Milk, we have said, leaves the stomach at rest; meat, on the contrary, keeps it in a necessary condition of excitement, which is useful with regard to digestion in general. If we prolong feeding on milk diet, we bring on a condition of weakness and apathy of the stomach, and it will easily be deranged by the slightest change of diet.

Meat, on the contrary, keeps up the activity of the stomach, comforts it, and in a general way facilitates the digestion. It is thus that, without considering substances with respect to their digestibility, it is important to know in what conditions their digestion takes place, in order that we may lay down the indications for hygiene and clinical practice.—*Med. and Surg. Reporter.*

